# Using Base-ml to Learn Classification of Common Vestibular Disorders on DizzyReg Registry Data

**DOI:** 10.3389/fneur.2021.681140

**Published:** 2021-08-02

**Authors:** Gerome Vivar, Ralf Strobl, Eva Grill, Nassir Navab, Andreas Zwergal, Seyed-Ahmad Ahmadi

**Affiliations:** ^1^German Center for Vertigo and Balance Disorders, University Hospital Munich, Ludwig-Maximilians-University, Munich, Germany; ^2^Computer Aided Medical Procedures, Department of Informatics, Technical University Munich, Munich, Germany; ^3^Department of Biometry and Epidemiology, Institute for Medical Information Processing, Biometry and Epidemiology, Ludwig-Maximilians-University, Munich, Germany; ^4^Department of Neurology, University Hospital Munich, Ludwig-Maximilians-University, Munich, Germany

**Keywords:** chronic vestibular disorders, classification, machine learning, multivariable statistics, clinical decision support (cdss), episodic vestibular symptoms

## Abstract

**Background:** Multivariable analyses (MVA) and machine learning (ML) applied on large datasets may have a high potential to provide clinical decision support in neuro-otology and reveal further avenues for vestibular research. To this end, we build base-ml, a comprehensive MVA/ML software tool, and applied it to three increasingly difficult clinical objectives in differentiation of common vestibular disorders, using data from a large prospective clinical patient registry (DizzyReg).

**Methods:** Base-ml features a full MVA/ML pipeline for classification of multimodal patient data, comprising tools for data loading and pre-processing; a stringent scheme for nested and stratified cross-validation including hyper-parameter optimization; a set of 11 classifiers, ranging from commonly used algorithms like logistic regression and random forests, to artificial neural network models, including a graph-based deep learning model which we recently proposed; a multi-faceted evaluation of classification metrics; tools from the domain of “Explainable AI” that illustrate the input distribution and a statistical analysis of the most important features identified by multiple classifiers.

**Results:** In the first clinical task, classification of the bilateral vestibular failure (*N* = 66) vs. functional dizziness (*N* = 346) was possible with a classification accuracy ranging up to 92.5% (Random Forest). In the second task, primary functional dizziness (*N* = 151) vs. secondary functional dizziness (following an organic vestibular syndrome) (*N* = 204), was classifiable with an accuracy ranging from 56.5 to 64.2% (k-nearest neighbors/logistic regression). The third task compared four episodic disorders, benign paroxysmal positional vertigo (*N* = 134), vestibular paroxysmia (*N* = 49), Menière disease (*N* = 142) and vestibular migraine (*N* = 215). Classification accuracy ranged between 25.9 and 50.4% (Naïve Bayes/Support Vector Machine). Recent (graph-) deep learning models classified well in all three tasks, but not significantly better than more traditional ML methods. Classifiers reliably identified clinically relevant features as most important toward classification.

**Conclusion:** The three clinical tasks yielded classification results that correlate with the clinical intuition regarding the difficulty of diagnosis. It is favorable to apply an array of MVA/ML algorithms rather than a single one, to avoid under-estimation of classification accuracy. Base-ml provides a systematic benchmarking of classifiers, with a standardized output of MVA/ML performance on clinical tasks. To alleviate re-implementation efforts, we provide base-ml as an open-source tool for the community.

## Introduction

Multivariable statistical analysis (MVA), and modern machine learning (ML) methods have the potential to serve as clinical decision support systems (CDSS) (1–3), including the computer-aided diagnosis (CADx) of vestibular disorders (4–8). In combination with large datasets and multi-site cohorts, MVA/ML classification algorithms allow for investigating interactions between patient variables, which is why recent works advocate that these methods should be used more widely in neuro-otology and vestibular neuroscience (9). However, there is a wide variety of MVA/ML methods available, and recent advances in deep learning (DL) with artificial neural networks (ANN) (10) add to the complexity of the field.

In this work, we followed three clinical three clinical scenarios in the differential diagnosis of vestibular disorders, and defined three respective classification problems with increasing difficulty. We applied a wide variety of MVA/ML/DL methods to investigate the suitability of automated classification for these clinical questions, and to compare the algorithmic outcomes with clinical expert intuition, both from the perspective of supposed task difficulty, and from the perspective of how the algorithms weighted feature importances toward diagnostic classification. For validation, we took advantage of the DizzyReg dataset, a large prospective registry of patients with vestibular disorders (11). The dataset is multimodal and contains three main categories of variables, namely patient characteristics, symptom characteristics, and quantitative parameters from vestibular function tests.

The first classification problem addresses two groups of patients, suffering either from bilateral damage to peripheral vestibular afferents (i.e., bilateral vestibular failure), or functional dizziness without evidence for relevant structural or functional vestibular deficits. Both syndromes present with the chief complaint of persistent dizziness. However, additional symptom features (e.g., triggers, extent of concomitant anxiety and discomfort) may vary considerably. We expected that machine learning can reliably differentiate both disorders based on patient characteristics (e.g., different age spectra), symptom characteristics, and vestibular function test (e.g., head impulse test or caloric testing).

The second classification task is, whether patients with primary functional dizziness (based on psychological triggers and stressors) can be separated against patients with secondary functional dizziness following a preceding organic vestibular disorder (such as acute unilateral vestibulopathy, or benign paroxysmal positional vertigo) (8). This setting is more complex, as patient and symptom characteristics may be similar, but the vestibular function tests may differ.

The third problem is directed to the differentiation of four episodic vestibular disorders, namely benign paroxysmal positional vertigo (BPPV), vestibular paroxysmia (VP), Menière disease (MD) and vestibular migraine (VM). This multi-class problem is supposed to be the most complex, because the demographic characteristics of patients and the spectrum of symptoms can be diverse and may overlap (e.g., between MD and VM), and vestibular function tests may be normal (e.g., in VP or VM).

To investigate classification on these three clinical objectives, we developed base-ml, a comprehensive test-bench for initial ML experimentation on clinical data. With this tool, we aim to provide clinical experts with a better intuitive feeling for the range of ML outcomes that can be expected on the given data. For better transparency, several methods can and should be investigated at the same time, subject to a comparable data pre-processing and cross-validation strategy. To this end, we compare several linear, non-linear and neural-network based ML algorithms, along with a novel graph deep learning method that we recently proposed (6, 12, 13). Following insights from multiple classification experiments for diagnostic decision support in our research over the last few years (4, 6, 13, 14), we also provide a multi-faceted analysis of algorithm outcomes, including an examination of class imbalance, multiple classification metrics, patient feature distributions, and feature importances as rated by the classifiers. To alleviate the implementation burden for multi-algorithm comparison and multivariate evaluation, we provide base-ml as an open-source tool^1^ to the vestibular research community, as a starting point for further studies in this direction.

## Materials and Methods

### DizzyReg Registry and Dataset

The objective of the DizzyReg patient registry is to provide a basis for epidemiological and clinical research on common and rare vertigo syndromes, to examine determinants of functioning and quality of life of patients, to identify candidate patients for future clinical research, to integrate information of the different apparative measurements into one data source, and to help understanding the etiology of the vestibular disorders.

The DizzyReg patient registry is an ongoing prospective clinical patient registry which collects all information currently stored in electronical health records and medical discharge letters to create a comprehensive clinical database of patient characteristics, symptoms, diagnostic procedures, diagnosis, therapy, and outcomes in patients with vertigo or dizziness (11). Study population includes patients with symptoms of vertigo and dizziness referred to the specialized out-patient center for vertigo and balance disorders. Recruitment into the registry commenced in December 2015 at the German Center for Vertigo and Balance Disorders (DSGZ), Munich University Hospital of the Ludwig-Maximilians-Universität. Inclusion criteria into the registry are symptoms of vertigo and dizziness, age 18 years and above, signed informed consent and sufficient knowledge of German.

Questionnaires were issued on first day of presentation to the study center to assess lifestyle and sociodemographic factors as well as self-reported perception of vertigo symptoms, attack duration and the time since first occurrence. Lifestyle and sociodemographic factors assessed using questionnaires include age, gender, education, physical activity, alcohol, smoking, sleep quality. The type of symptoms of patients included: vertigo, dizziness, postural instability, problems while walking, blurred vision, double vision, impaired vision, nausea, vomiting. Concomitant ontological or neurological symptom are documented with a focus on otological symptoms, i.e., hearing loss, tinnitus, aural fullness, pressure, hyperakusis, and neurological symptoms, i.e., headache, type of headache, photo-/phonophobia, double vision, other symptoms (ataxia, sensory loss, paresis, aphasia).

The evolution of symptoms was reconstructed by the frequency and duration of attacks. All aspects of history taking in the DizzyReg follow established concepts such as “So stoned” (15), the “Five Keys” (16) and the “Eight questions” (17). Frequency or time of onset of symptoms was included as a categorial variable with the following categories: “less than 3 month,” “3 months to 2 years,” “more than 2 years,” “more than 5 years,” and “more than 10 years.” The duration of symptoms is registered in the categories “seconds to minutes,” “minutes to hours,” “hours to days,” “days to weeks,” “weeks to months,” “continuous.”

The registry further collects information on symptoms, quality of life (EQ5D) and functioning (DHI and VAP) in a few standardized questionnaires. Information on triggers is gathered by the respective categories of the Dizziness Handicap Inventory and by elements of the Vertigo Activity and Participation Questionnaire (VAP) (e.g., head movement, position change, physical activity etc).

#### DHI

The Dizziness Handicap Inventory (DHI) is a well-known and widely used measure to assess self-perceived limitations posed by vertigo and dizziness (18). A total of 25 questions are used to evaluate functional, physical, and emotional aspects of disability. Total score ranging from 0 to 100 is derived from the sum of responses (0 = No, 2 = sometimes, 4 = Yes).

#### Quality of Life

Health-related quality of life was assessed with the generic EuroQol five-dimensional questionnaire (EQ-5D-3L). This is subdivided into five health state dimensions namely mobility, self-care, usual activities, pain/discomfort, and anxiety/depression, with each dimension assessed in three levels: no problem, some problem, extreme problems. These health states were converted into EQ5D scores using the German time trade-off scoring algorithm (19). The resulting total EQ5D score ranges from 0 to 1 with higher scores indicating better quality of life.

#### Vertigo Activity and Participation Questionnaire (VAP)

Functioning and participation were assessed based on the Vertigo Activity and Participation Questionnaire (VAP). The VAP is specifically designed for persons with Vertigo and Dizziness and can be used for people of different countries (20–22). The VAP measures functioning and participation in two scales consisting of six items each. Using weights derived from Rasch analysis the first scale has a range of 0–23 points and the second of 0–20 points with higher scores indicating more restrictions.

Data protection clearance and institutional review board approval has been obtained (Nr. 414-15).

### Classification Tasks and Cohorts

As mentioned in the introduction, three classification problems with increasing complexity were tested: (1) bilateral vestibular failure vs. functional dizziness; (2) primary vs. secondary functional dizziness; (3) BPPV vs. VP vs. MD vs. VM. Table 1 provides information about the group cohorts for each task.

**Table 1:** Clinical tasks with respective classes of chronic/episodic vestibular disorders, and respective cohort details.

	**Diagnosis abbreviation**	***N***	**Age mean (s.d.)**	**EQ5D**	**DHI**	**Female/Male**
**Task 1**						
Bilateral vestibular failure	BVF	66	65.0 (17.0)	0.8 (0.2)	46.2 (22.6)	27/39
Functional dizziness	FD	346	47.2 (14.5)	0.8 (0.2)	43.3 (18.4)	178/168
**Task 2**						
Functional dizziness (Secondary)	FDS	204	52.1 (14.7)	0.8 (0.2)	48.0 (18.8)	130/74
Functional dizziness (Primary)	FDP	151	45.4 (14.6)	0.8 (0.2)	42.6 (17.6)	77/74
**Task 3**						
Benign Parox. Pos. Vertigo	BPPV	134	57.0 (12.1)	0.8 (0.2)	45.0 (19.6)	88/46
Menière disease	MM	142	53.4 (13.3)	0.9 (0.2)	43.9 (19.8)	78/64
Vestibular migraine	VM	215	44.5 (14.0)	0.8 (0.2)	41.8 (18.6)	145/70
Vestibular paroxysmia	VP	49	51.6 (14.2)	0.9 (0.2)	38.8 (22.5)	20/29

### Classification Pipeline

**Figure 1:**
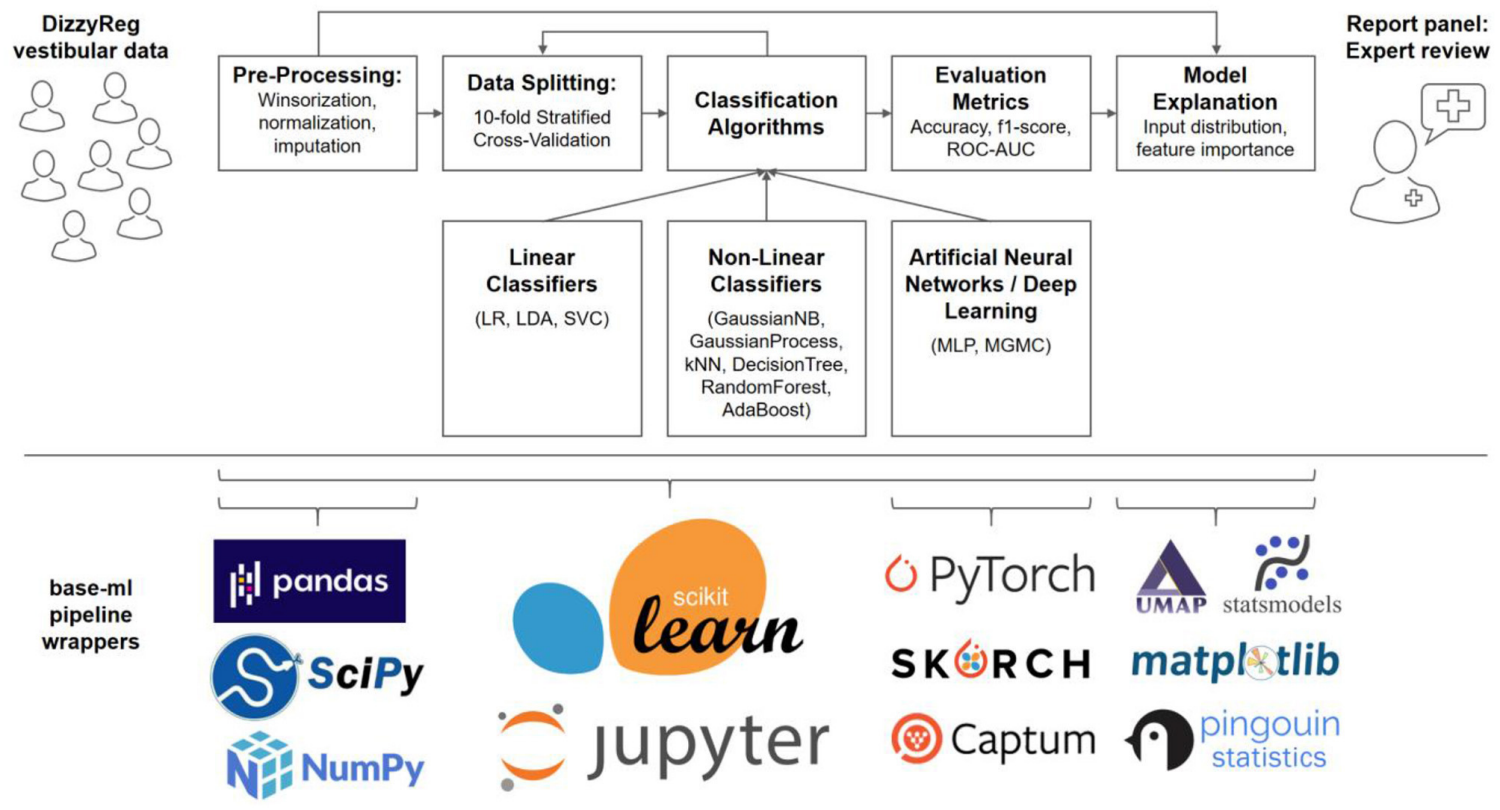
Components and methods of the classification workflow applied to vestibular data in DizzyReg. Raw tabular data is pre-processed and split into 10-folds for stratified cross-validation and for estimation of prospective classification performance. Various linear, non-linear and neural classifiers are repeatedly trained on all folds in the data, the evaluation is performed with various classification metrics. The metrics, along with model explanations, are presented to experts in form of a report panel, who can review the classification outcome and model performance. All pipeline components are implemented in base-ml, a comprehensive software tool which we provide open-source to the vestibular community as a starting point for similar studies. Implemented in Python, and centered around scikit-learn, it comprises various modules for data science, machine learning, descriptive statistics, explainable AI and visualization. Details on base-ml are described in section Base-ml Framework.

A typical machine learning pipeline comprises several steps that interplay toward a high-accuracy prediction (23). After data import, a set of pre-processing routines are applied to patient features, before data is split into several folds for training and testing, using one or several classification algorithms. The classifier performance is evaluated using several quantitative metrics, and finally presented and explained to a clinical expert on vestibular disorders, for a critical review. Figure 1 presents an overview of our methodological pipeline in this work.

#### Pre-processing

Multimodal medical datasets commonly pose several challenges for CADx algorithms, including noisy or missing patient features with spurious outliers (24–26), a mixture of categorical and continuous variables (27), and different statistical distribution of variables (23). To account for outliers and different data ranges in DizzyReg variables with continuous distributions, we perform a 90% winsorization which sets extreme values to the 5th and 95th percentiles, before applying a z-transformation (27) which normalizes all variables into a comparable zero-mean and unit-variance data range. Categorical variables are binarized where possible, or represented in form of a one-hot encoding (a.k.a. one-of-K encoding), which creates a binary column for each category and sparsely represents the categories with a value of 1 in the respective column and 0 in all the other columns. To account for missing values, we perform a mean-imputation (24) if <50% of values are missing in the population, otherwise the feature is omitted from the patient representation.

#### Data Splitting

In predictive statistics, in particular in the machine learning community, it is common to assess the prediction performance via hold-out test datasets, which are often randomly sampled and kept separate from the training dataset until the time of pseudo-prospective evaluation (27). Sampling a single test set could result in a biased selection and thus in an overly optimistic or pessimistic test evaluation. To avoid this, it is recommendable to evaluate with multiple test sets, which are sampled either through random shuffling, or through a k-fold splitting. Following common recommendations, we set k to 10 in this work (28). This yields exactly one prediction for each subject in DizzyReg, and exactly ten estimates for the prospective classification performance of each classifier. As recommended by Kohavi in (29), we additionally apply a stratified cross-validation to make sure that each fold has approximately the same percentage of subjects from each class, which is important especially in the case of class imbalance in the dataset. To ensure that individual classifiers are being trained in a suitable parametrization, we additionally perform hyper-parameter optimization using random search, in a nested cross-validation setup (for details, see section Appendix C).

#### Classification Algorithms and Metrics

Intuitively, ML classifiers try to assign class labels to samples (e.g., patients, represented as multivariable numerical vectors), by fitting separation boundaries between classes in high-dimensional space. Mathematically, these boundaries are expressed in form of a classification function = *f*(*x*), which separate the statistical distributions of classes *C* in the input space *X*. The past decades of ML research have yielded a diverse set of mathematical models for separation boundaries, and algorithms to fit them to a set of training data *X*, including linear regression boundaries, rule-based, instance-based, tree-based, kernel-based or Bayesian methods (23), as well as the recent renaissance of artificial neural networks and deep learning (10). Importantly, no single method is guaranteed to perform best on all datasets (30), which is why it is recommendable to test multiple algorithms and let their performances be compared and critically reviewed by a domain expert, instead of deciding on a single algorithm a priori. Therefore, as described in the introduction, we compare several linear, non-linear and neural-network based ML algorithms, along with a novel graph deep learning method that we recently proposed (6, 12, 13). Details on all classifier models and their parametrization are given in section Overview of Selected Classification Algorithms. We quantitatively evaluate the classification performance with three metrics: area-under-the-curve of a receiver-operating-characteristic (ROC-AUC), as well as accuracy and f1-score, defined as (TP/TN/FP/FN denote true or false positives or negatives):

$$\begin{array}{lll} \text{Accuracy} & = & \;\frac{TP+TN}{N};\text{ f}1-\text{score}=\frac{2\;Prec\;Rec}{Prec+Rec};\\ Prec & = & \frac{TP}{TP+FP};\;Rec=\frac{TP}{TP+FN} \end{array}$$

#### Model Explanation

A necessary tradeoff in predictive statistics and ML is to choose between model accuracy and model interpretability (31). While linear methods like logistic regression are typically more interpretable, non-linear models, depending on their complexity, are often compared to black boxes. By now, however, “Explainable AI” is a dedicated branch in ML research, and numerous model-specific and model-agnostic methods are available that can partially explain ML prediction outcomes (32). Two common ways to explain model performance is to analyze the distribution of input samples (4, 33), and to analyze feature importance (34), especially in a clinical setting (35).

First, we perform a non-linear mapping of the *d*-dimensional input distribution after pre-processing onto the 2D plane, and we visualize whether class distributions were already visible in the input data, or whether the input data distribution has unexpected or undesired properties, a technique which has been elucidating in our research before, e.g., in the mapping of posturography data (4). To this end, we utilize “Uniform Manifold Approximation and Projection” (UMAP) (33), a topology-preserving manifold learning technique for visualization and general non-linear dimensionality reduction.

Second, we analyze which patient features contributed to classification outcomes the most, which is a clinically interesting aspect of classifiers. We obtain the “feature importances” for non-ANN-based models and “feature attributions” for ANN-based models. For linear classifiers (see section Linear Classifiers), these can be obtained through the model coefficients (27). For non-linear classifiers (see section Non-linear Classifiers), such as tree-based models, we obtain their feature importance using the Gini-impurity criterion (36). For neural-network based models such as MLP and MGMC (see section Neural Network and Deep Learning Classifiers), we use the Integrated Gradients algorithm (37) and calculate the feature importance by taking the feature attributions of every sample in the training dataset toward their respective ground truth class labels. Obviously, not every classification algorithm yields the same ranking for feature importances. It is argued that a combination of several feature importance rankings can provide more reliable and trustworthy (34). Therefore, for our report to the expert, we aim at presenting a single table with the top 10 most important features for the given classification problem. To merge the feature importance rankings of the different classifiers into a single list, we propose and apply a heuristic for Relative Aggregation of Feature Importance (RAFI), which comprises the following three steps. First, we take the absolute values of all feature importances, to account for algorithms with negative weights (e.g., negative coefficients in linear regression). Second, we normalize the range of importance scores across different classifiers, by computing the percentual importance. Third, we aggregate all normalized global importances by summation, and report the top 10 most important features across all classifiers to the experts for review. In detail, for each feature φ_*i*_ (*i ϵ* [1, …, *d*]), and across *F* different classifiers, each with feature importances *I*_*j*_(φ_*i*_) (*j ϵ* [1, …, *F*]), we calculate the global feature importance *I*_0_(φ_*i*_) as follows:

$$\begin{array}{l} I_{0}(\varphi_{i})=\sum_{j=1}^{F}\frac{abs(I_{j}(\varphi_{i})\;)}{\overset{d}{\underset{i=1}{\sum }}abs(I_{j}(\varphi_{i})\;)\;} \end{array}$$

### Overview of Selected Classification Algorithms

In this work, we apply and compare the outcomes for a total of 11 classification methods, which we chose to represent a wide range of algorithmic approaches. This collection is larger than what is typically encountered in CDSS research, as mentioned, to provide the expert with a better intuitive feeling for the range of outcomes that can be expected on the given data. The algorithms are grouped into three general categories: linear, non-linear, and ANN-based classifiers. Since explaining the inner workings of all methods in detail is out of scope for this work, each algorithm will be outlined only briefly in the following, with its most important parametrizations (if any), and a reference to explanatory material for the interested reader.

#### Linear Classifiers

As linear classifiers, we apply *Linear Discriminant Analysis (LDA), Logistic Regression (LR)* and *Support Vector Classifiers (SVC)*. All three methods try to fit a set of linear hyperplanes between the *d*-dimensional distributions of the classes. *LDA* [(19), chapter 4.3] models the distribution for each class with a Gaussian and calculates the probability of belonging to a class as the maximum posterior probability in a Bayesian manner. We apply LDA in a default parametrization, without additional regularizations such as shrinkage. *LR* [(19), chapter 4.4] directly learns the posterior distribution of the target class and models it using a sigmoid-activated linear function. We apply LR with simple L2 regularization to avoid overfitting the parameters of the model on the training set. *SVC* (38) is a support-vector machine (SVM) with a linear kernel, which learns a hyperplane that maximizes the gap between the classes, giving slack to key samples (“support vectors”) to account for class overlap in the joint distribution. To avoid overfitting, we apply a standard squared l2 penalty term using a regularization parameter of 0.25.

#### Non-linear Classifiers

##### Gaussian Naïve Bayes (GNB)

GNB [(19), chapter 6.6.3] is a variant of Naïve Bayes (NB) that allows continuous input features, under the assumption of Gaussian distribution and mutual independence. Class posterior probabilities for new samples are calculated using Bayes Rule. We parametrize *GNB* to estimate class prior probabilities directly from training data, rather than imposing them a-priori.

##### Gaussian Process Classifier (GP)

GP (39) are a Bayesian alternative to kernel methods like non-linear SVMs. In classification, it models and approximates the class posterior probability as a Gaussian distribution. We set the initial kernel used for GP fitting to a zero-mean, unit-variance radial basis function (RBF), which is then refined during the fitting to training data.

##### K-Nearest Neighbors Classifier (KNN)

KNN [(19), chapter 2.3.2] classification is an instance-based method, where a sample's class is determined by the majority class label vote of the sample's k-nearest neighbors. We compute similarity as Euclidean distance between two patients' feature vectors, and we use 10 nearest neighbors in the training set to predict the class label of a test input.

##### Decision Tree Classifier (DT)

DT (36) are a form of rule-based classifiers. A tree represents a hierarchical set of rules or decisions, each decision splitting the feature space in a single feature dimension, using an optimal splitting threshold which is calculated using information-theoretic criteria. Each new sample is passed down the tree, following splitting rules, until a leaf is hit in which a class distribution and majority class is stored. In this work, we use trees with Gini impurity as the splitting criterion, and we allow trees to expand up to a maximum depth of five.

##### Random Forest Classifier (RF)

RF (40) are an ensemble of multiple decision trees, where each tree is trained using a random subset of training data and a random subset of features. Due to the randomization, the individual trees are highly uncorrelated. Therefore, the ensemble output, which is calculated as an average vote from all trees, weighted by their confidences, is highly robust against various data challenges, such as high dimensional input spaces, noisy data, or highly different data distributions across variables. In this work, we use an ensemble of 10 trees, each with a maximum depth of 5 decision levels.

##### Adaptive Boosting Classifier (AB)

AB (41), similar to RF, is another ensemble method that combines multiple “weak” classifiers in order to form a much “stronger” classifier. A key difference is the boosting mechanism, i.e., the ensemble is allowed to iteratively add new weak classifiers, which are trained with a higher weight on those input instances that are still being misclassified. In this work, we use decision stubs (i.e., decision trees with a depth of (1) as the weak base classifiers, and we allow the maximum number of classifiers to reach up to 50.

#### Neural Network and Deep Learning Classifiers

##### Multi-Layer Perceptron (MLP)

MLP [(19), chapter 11] consider input features as activated neurons followed by one or several fully connected layers (so-called hidden layers) of artificial neurons which weight and sum incoming neuronal connections, before applying a non-linear activation function. The network weights are estimated using the backpropagation algorithm. In this work, we parametrized an ANN with two hidden layers (64 and 32 neurons), and protect every layer against overfitting, as is commonly achieved by applying dropout (*p* = 0.3) (42), followed by batch normalization (43).

##### Multi-Graph Geometric Matrix Completion (MGMC)

MGMC (13) is a graph-based neural network (GNN) model which we proposed recently, as an extension to our previously published geometric matrix completion approach for multimodal CADx (12). It models the classification problem as a transductive geometric matrix completion problem. Importantly, MGMC is designed to deal with the common problem of missing values in large medical datasets (25), by simultaneously learning an optimal imputation of missing values, along with the optimal classification of patients. MGMC models the patients as nodes in a graph, and computes the edges in the graph through a similarity metric between patients. The similarity is based on a few meta-features (e.g., sex, age, genetic markers etc.), which allows MGMC to span a graph between patients akin to a social network. In previous works, GNNs have shown promising results and a complementary approach in the field of CADx. In this work, we compute multiple patient graphs, each based on similarity measures of a single meta-feature, namely gender (same gender), age (age difference ± 6 years), EQ5D score (score difference of ± 0.06), and DHI score (score difference of ± 11). As advanced model parameters, we use five timesteps for the recurrent graph convolutional network, Chebyshev Polynomials of order five, and a single hidden layer before the output (16, 32, or 64 neurons, depending on the classification task).

### Statistical Methods

The most important features detected by RAFI (cf. section Classification Pipeline) are presented for expert review and interpretation. Each of these features is compared across patient classes via hypothesis tests, to provide a first glance whether there are significant differences across groups. For continuous variables, and in the case of two classes, we first test each variable for normal distribution in each of the patient group with a Shapiro-Wilk test (44). If so, we apply an unpaired two-tailed *t*-test (27), if not, we apply a Mann-Whitney U test (45). For more than two classes, we apply a one-way ANOVA test (27), or a Kruskal-Wallis (46) as an alternative for non-parametric testing, and report the group-level *p*-value. For categorical values, we apply a Chi-squared independence test (47). We report *p*-values for hypothesis tests on all variables, and assume significance at an alpha-level of *p* < 0.05.

### Base-ml Framework

As described in the previous sections Classification Pipeline-Statistical Methods numerous methods are necessary to imple1ment a full data science and machine learning pipeline, for a multimodal clinical problem like vestibular classification, and in a multi-site dataset like DizzyReg. Naturally, re-implementing this stack of methods is a time-consuming effort, which should ideally be avoided across research groups. To alleviate future classification experiments similar to this work, and to provide the community with a starting point, we developed base-ml, an open-source Python package^1^ provided by the German Center of Vertigo and Balance Disorders. The package can enable a rapid evaluation of machine learning models for prototyping or research. As illustrated in Figure 1 (lower panel), it is built around scikit-learn (48) as a backbone, which is a reference toolkit for state-of-the-art machine learning and datascience. We complement scikit-learn with various Python modules: *pandas* (49) for data IO and analysis; scipy and numpy (50) for fast linear algebra on array-shaped data; *PyTorch* (51) for implementation of ANNs and more advanced deep learning models like MGMC; *skorch*^2^ for integration of PyTorch models into the scikit-learn ecosystem; the Captum^3^ library for model interpretability and understanding, which we use for calculation of feature importance in ANNs using Integrated Gradients (37); UMAP (33) for non-linear 2D mapping and visualization of the patients' input distribution; statsmodels (52) and pingouin (53), two Python libraries for descriptive statistics and hypothesis testing; and matplotlib for plotting and scientific visualization. Importantly, using skorch, we enable potential adopters of base-ml to integrate both inductive and transductive neural training workflows and even deep learning models into a comparative benchmark with more traditional ML methods. Skorch combines the ease of use of scikit-learn training workflows and PyTorch's GPU-enabled neural network models. In addition, with base-ml, one can easily evaluate graph-based neural network models.

## Results

The following sections reproduce the classification reports produced by base-ml on the three clinical tasks described in the introduction. It is important to note that base-ml is not restricted to vestibular classification scenarios. As a sanity check for base-ml, regarding classification outcomes, and comparability to baseline results in literature, we perform two additional experiments. Those two base-ml experiments are performed on non-vestibular datasets, i.e., one artificially generated dataset, and one Alzheimer's disease classification dataset, which has been widely studied in literature. To keep the main body of this manuscript dedicated to vestibular analysis, we report on non-vestibular results in the Appendix.

### Results on Task 1 (Bilateral Vestibular Failure vs. Functional Dizziness)

**Figure 2:**
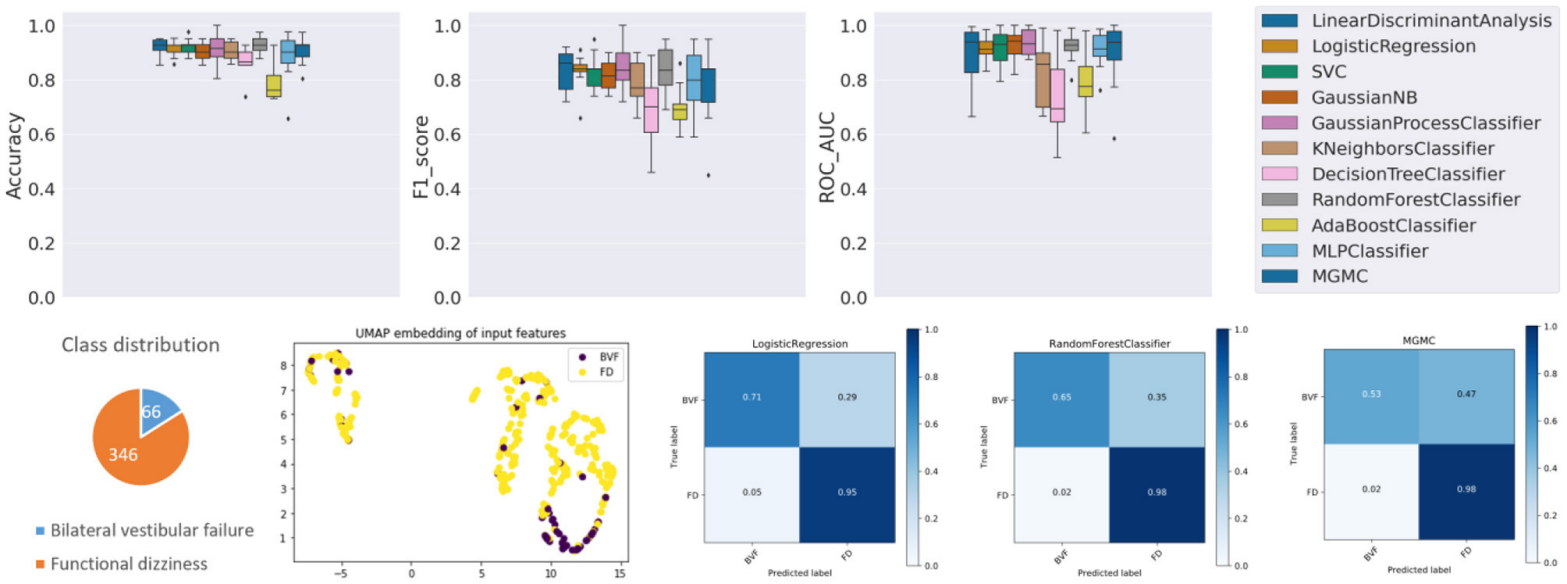
Results panel produced by base-ml for Task 1. It comprises: boxplots for the three classification metrics, accuracy, f1-score and ROC-AUC; a pie chart to highlight potential class imbalances for this task; the UMAP 2D embedding of all patients' input feature vectors; and a more detailed overview of classification outcomes in form of confusion matrices, for three classifiers, LR, MGMC and RF.

The results panel for this classification task, as produced by the base-ml framework, is visible in Figure 2. The boxplots with metrics illustrate a wide range of classification performances for all classifiers, with an accuracy over the 10 folds between 78.7% ± 6.4% (AdaBoost) and 93.0% ± 3.5% (RF), an f1-score between 0.683 ± 0.144 (DecisionTree) and 0.848 ± 0.091 (GaussianProcess), and an average ROC-AUC between 0.727 ± 0.145 (DecisionTree) and 0.937 ± 0.050 (GaussianProcess), followed closely by a ROC-AUC of 0.921 ± 0.056 (RF). Quantitatively, Gaussian Process classifiers are the top-performing model on this task, and slightly outperform the best-performing neural network model MGMC (mean accuracy/f1-score/ROC-AUC: 90.8%/0.782/0.893). In fact, on this task, even one of the best linear models, LR, performs better than MGMC and almost as good as RF (mean accuracy/f1-score/ROC-AUC: 91.3%/0.831/0.917). The confusion matrices reveal that the group with functional dizziness was detected with a very high sensitivity between 95% (LR) and 98% (MGMC/RF), compared to a much lower sensitivity between 53% (MGMC) and 71% (LR) for patients with bilateral vestibular failure. Notably, hyper-parameter optimization had a positive effect on the outcomes of Task 1, and the average accuracy of all classifiers increased from 87.0 to 89.6% after parameter tuning.

Regarding class imbalance, which is important to consider in context with classification performance, the pie chart (cf. Figure 2, bottom left) shows that BVF is strongly under-represented in this DizzyReg subset, at 66 vs. 346 patient samples (16.0% of patients). Finally, the UMAP embedding shows that the FV subjects (colored in yellow) are already clustered and topologically separated from the BVF subjects (colored in purple) at the level of normalized input data. This underlines that the patients have clearly separate characteristics at a feature level, and classifiers have a good chance at fitting decision boundaries between the two groups. The UMAP plot reveals another interesting point, namely that the input data is clearly separated into two clusters, the implications of which are discussed below.

The base-ml output also produces Table 2, with feature importance scores aggregated with the RAFI heuristic (cf. section Classification Pipeline). Among the top ten features, six features are related to (Video-) Head Impulse Testing (HIT/vHIT; HIT left/right abnormal, vHIT normal result, vHIT gain left/right) or caloric testing, all of which are also statistically significantly different between the two groups at a level of *p* < 0.001. The most important feature is patient age, also with a significantly different expression between the two groups (63.8 ± 15.6 vs. 47.3 ± 14.1 years, *p* < 0.0001). The remaining three features are related to subjective judgement of disability by patients, namely the depression score in EQ5D (*p* < 0.001), a perceived handicap in DHI (*p* < 0.01), and the actual perceived health condition (*p* = 0.133).

**Table 2:** Top 10 most important features in Task 1, aggregated over multiple classifiers.

**Rank**	**Feature**	**Feature Type**	**Bilateral vestibular failure**	**Functional dizziness**	***P*-Value**
1	Age (yrs)	Questionnaire	63.83 ± 15.64	47.33 ± 14.12	<0.0001
2	HIT: right, abnormal	Neurological investigation P1	77.40%	3.40%	<0.0001
3	HIT: left, abnormal	Neurological investigation P1	77.40%	2.30%	<0.0001
4	vHIT: normal result	Apparative tests	14.30%	92.20%	<0.0001
5	vHIT: gain left	Apparative tests	0.8 ± 0.04	0.97 ± 0.12	<0.0001
6	EQ5D: fear, depression	Questionnaire	28.60%	66.40%	<0.0001
7	Caloric: normal result	Apparative tests	31.90%	91.80%	<0.0001
8	vHIT: gain right	Apparative tests	0.71 ± 0.09	0.92 ± 0.15	<0.001
9	DHI: Q21, perceived handicap	DHI	81.20%	92.60%	<0.01
10	LIFEQ: Q7, Actual perceived health condition	LIFEQ	62.51 ± 18.48	58.11 ± 18.9	0.133

### Results on Task 2 (Primary vs. Secondary Functional Dizziness)

**Figure 3:**
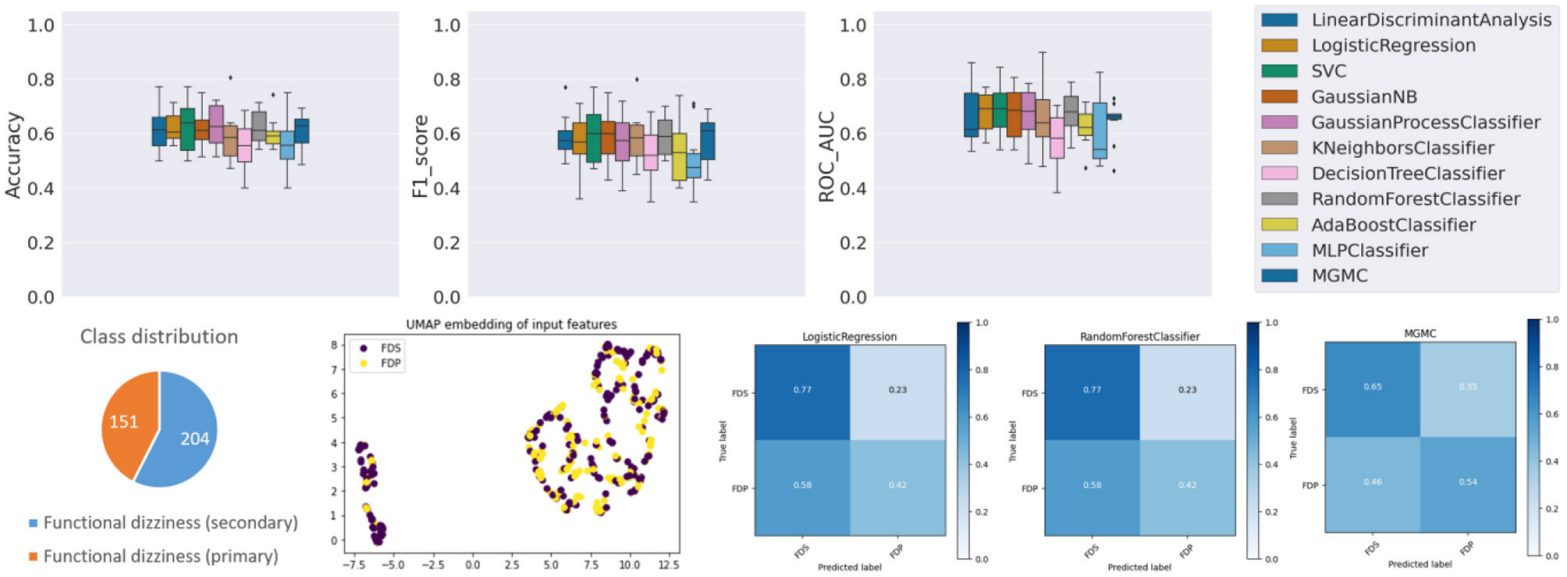
Results panel produced by base-ml for Task 2.

Compared to task 1, the performance of the 11 classifiers on task 2 is more homogeneous (cf. Figure 3), i.e., all classifiers classify with a within a similar accuracy range between 55.2% (DecisionTree) and 62.8% (GaussianProcess), a f1-score range between 0.498 (MLP) and 0.596 (SVC), and ROC-AUC range between 0.571 (DecisionTree) and 0.689 (SVC). Overall, this classification task is dominated by the linear classification algorithm SVC and the non-linear GaussianProcess classifiers, while the DecisionTree and neural network classifier MLP/ANN are the worst-performing algorithms in terms of accuracy and f1-score. The graph neural network method MGMC and RF had an accuracy of 60.6 and 62.2%, both are close to the average accuracy of all classifiers (60.4%). The confusion matrices reveal that LR and RF have an equally high sensitivity for secondary functional dizziness (77%), compared to MGMC (65%), but a comparably lower sensitivity for primary function dizziness (LR/RF: 42%, MGMC: 54%). Notably, hyper-parameter optimization had very little effect on the outcomes of Task 2, as the average accuracy of all classifiers stayed at 60.4% both with and without the parameter tuning.

Again, the lower classification performance could partly be due to class imbalance, i.e., a slight underrepresentation of primary functional dizziness in this DizzyReg subset (42.5% primary vs. 57.5% secondary), however the class imbalance is not as severe as in task 1. The UMAP feature embedding shows that after pre-processing, two clearly separated clusters emerge in the topology of the data. Again, the source for this data separation is not clear and will be discussed further below. However, in the smaller cluster, most of patients are from the group with secondary functional dizziness (purple points), while in the larger cluster, there is a mix of both groups, and this mix is not clearly separable by data topology alone. The classification algorithms still can achieve a certain level of data separability in high-dimensional space, but it is noteworthy that the UMAP embedding reflects that task 2 is more challenging compared to task 1, even before the classifiers are applied.

The top 10 most important features for task 2 (cf. Table 3) are largely different from task 1. Expectedly, a normal caloric result (rank 1) and the vHIT gain left/right (ranks 4 and 2) and abnormal HIT result on the right (rank 9) differ in both groups. Patients with primary functional dizziness are younger (rank 3) and tend to drink more alcohol (≥1 drink in the last week, rank 6). One item from the DHI plays an important role for separation, related to problems turning over while in bed (rank 7), and another life quality factor, LIFEQ Q7, i.e., the actual perceived health condition, is relevant as well (rank 8). The duration of vertigo is important as well, in particular whether the duration is between 20 and 60 min (rank 6). Finally, the depression/fear score in the EQ5D questionnaire is relevant (rank 10). All features except EQ5D fear/depression and LIFEQ Q7 are significantly different between the two groups. It is important to note though that multivariable classifiers do not need to depend on univariate feature significance. In high-dimensional space, these two univariately non-significant features may still contribute to a better separation boundary.

**Table 3:** Top 10 most important features in Task 2, aggregated over multiple classifiers.

**Rank**	**Feature**	**Feature type**	**Functional dizziness (secondary)**	**Functional dizziness (primary)**	***P*-Value**
1	Caloric: normal result	Apparative tests	73.10%	96.20%	<0.0001
2	vHIT: gain right	Apparative tests	0.87 ± 0.18	0.92 ± 0.19	<0.0001
3	Age (yrs)	Questionnaire	51.79 ± 13.91	45.61 ± 14.21	<0.0001
4	vHIT: gain left	Apparative tests	0.92 ± 0.13	0.97 ± 0.12	<0.0001
5	Vertigo time: 20–60 min	Questionnaire	13.20%	5.30%	<0.05
6	>= 1 alcoholic drink last week	Questionnaire	43.60%	58.30%	<0.01
7	DHI: Q13, problems turning over in bed	DHI	43.80%	25.70%	<0.001
8	LIFEQ: Q7, Actual perceived health condition	LIFEQ	57.28 ± 19.61	59.34 ± 18.53	0.111
9	HIT: right, abnormal	Neurological investigation P1	13.70%	1.40%	<0.0005
10	EQ5D: fear, depression	Questionnaire	60.0%	70.0%	0.069

### Results on Task 3 (BPPV vs. VP vs. MD vs. VM)

**Figure 4:**
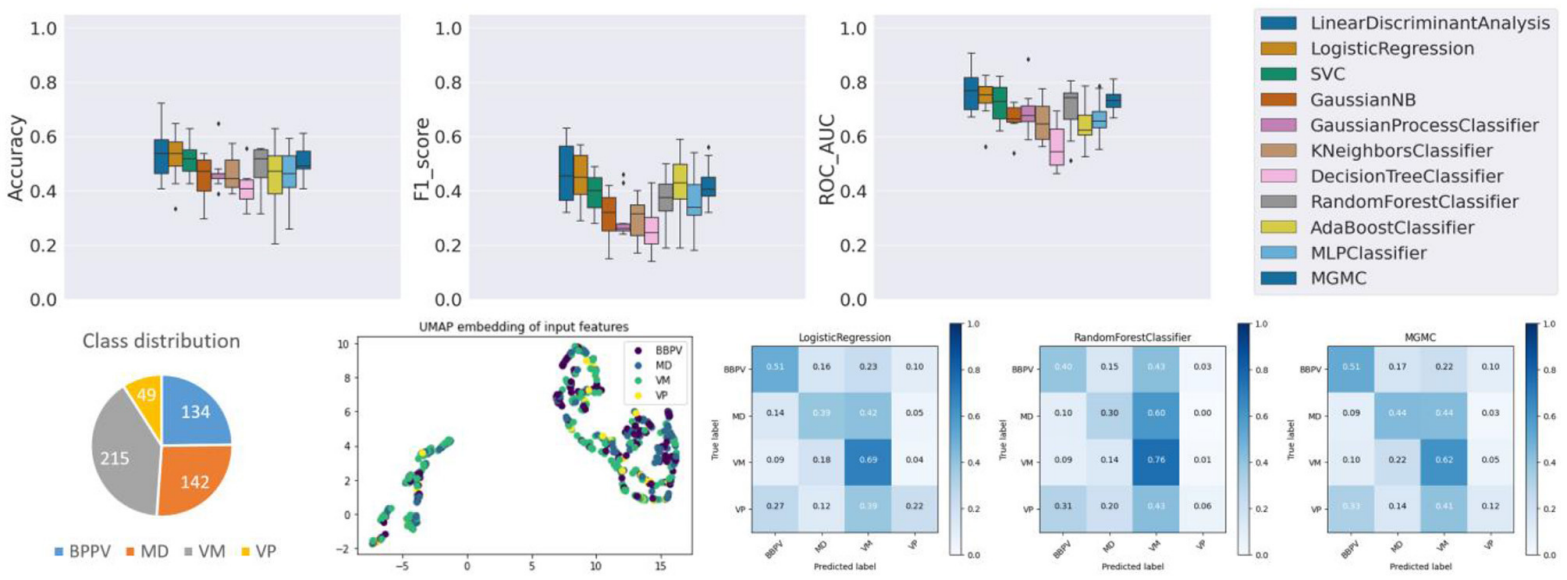
Results panel produced by base-ml for Task 3.

Already at first glance (cf. Figure 4), and as clinical intuition suggested, task 3 is the most challenging of the three classification tasks. Compared to the average classifier accuracy of task 1 (89.6%) and task 2 (60.4%), the accuracy on task 3 is much lower (48.0%). Individually, the classifiers have an accuracy range between 40.6% (DecisionTree) and 54.3% (LDA), a f1-score range between 0.269 (DecisionTree) and 0.461 (LDA), and a ROC-AUC range between 0.564 (DecisionTree) and 0.764 (LDA). Overall on task 3, linear classifiers, and LDA in particular, classify with the highest accuracy. The RF classifier, on the other hand, only has an average performance on task 3 (accuracy/f1-score/ROC-AUC: 48.5%/0.372/0.702), in comparison to tasks 1 and 2. The confusion matrices reveal that the disorders VM, BPPV, MD and VP can be classified with a decreasing order of classification sensitivity (e.g., for LR approximately: 70%, 50%, 40%, 20%). On task 3, hyper-parameter optimization had a much higher effect on the classifierd outcomes than in tasks 1 and 2, i.e., after parameter tuning, the average classification accuracy of all models increased from 44.2 to 48.0%.

Class imbalance probably plays a role here as well, as this ordering almost coincides with the class representation in the dataset (VM: 39.8%, BPPV: 24.8%, MD: 26.3%, VP: 9.1%). Looking at the UMAP embedding, the same separation of the data cloud into two clusters is clearly visible, and the four episodic vestibular disorders are visually not clearly separable within the two clusters, which again anticipates the difficulty of the classification task.

Regarding the 10 most important features (cf. Table 4), mean patient age ranks on the top (BPPV oldest, VM youngest). Second most important is vertigo time <2 min (which is most frequent in BPPV and VP). Expectedly, several features are related to body relocation, e.g., problems getting into, out of, or turning over inside the bed (DHI Q13, rank 3; VAP Q2, rank 4), bending over (DHI Q25, rank 7), or vertical climbing (VAP Q7, rank 10). Accompanying headache is ranked in 6th position and indicative for VM. There is only one apparative feature relevant for task 3 (normal caloric test, rank 5), with MD being the only group with relevantly abnormal results.

**Table 4:** Top 10 most important features in Task 3, aggregated over multiple classifiers.

**Rank**	**Feature**	**Feature type**	**BBPV**	**MD**	**VM**	**VP**	***P*-Value**
1	Age (yrs)	Questionnaire	56.6 ± 11.4	53.3 ± 13.0	44.7 ± 13.3	51.6 ± 13.6	<0.0001
2	Vertigo time: <2 min	Questionnaire	44.80%	12.70%	17.20%	71.40%	<0.0001
3	DHI: Q13, problems turning over in bed	DHI	87.90%	47.50%	44.20%	34.70%	<0.0001
4	VAP: Q2, problems to get in/out/turn over in bed.	VAP	93.30%	68.60%	58.50%	49.00%	<0.0001
5	Caloric: normal result	Apparative tests	85.90%	49.50%	84.80%	100.00%	<0.0001
6	Accompanying headache	Questionnaire	16.80%	19.00%	53.50%	15.00%	<0.0001
7	DHI: Q25, bending over increases problems	DHI	76.10%	60.30%	61.20%	61.20%	<0.05
8	DHI: Q6, restricted participation in social activities	DHI	71.40%	82.90%	75.50%	65.30%	<0.05
9	DHI: Q22, increased stress on family/friend relationships	DHI	23.10%	48.90%	45.60%	38.80%	<0.0001
10	VAP: Q7, Vertical climbing (stairs/lift)	VAP	60.00%	64.90%	62.00%	45.70%	0.139

## Discussion

In this paper, we have described several approaches for multivariable analysis and machine learning classification of three different patient cohorts from the vestibular registry dataset DizzyReg, i.e., functional dizziness vs. bilateral vestibular failure, primary vs. secondary functional dizziness, and BPPV vs. Meniére's disease vs. vestibular migraine vs. vestibular paroxysmia. Clinically, the three tasks were rated with an increasing difficulty and the machine learning classifier performances reflected this grading, with an average accuracy of 87.0, 60.5, and 44.3%, respectively. Using results produced by base-ml, we put these accuracy scores into context with class imbalance, input feature embedddings, confusion matrices and sensitivity scores, as well as tables with the top 10 most important features, aggregated over several classifiers using the proposed RAFI heuristic. In the following, we are going to discuss these results, both from a technical and clinical perspective.

### Technical Aspects

The results of the three classification experiments highlight several important points. We believe it to be apparent from the results that it is beneficial to run and benchmark several classification algorithms, ideally from different categories, such as linear, non-linear and neural models. Even a supposedly easy task from a medical perspective does not necessarily lead to a matching classifier performance, depending on which model is used (e.g., 78% classification accuracy in task 1 with Naïve Bayes), hence an a-priori selection could result in too pessimistic an assessment of classification potential using machine learning. Therefore, a wide range of methods in one comprehensive framework might benefit research groups that are new to the field of ML on clinical data. Further, linear models should always be tested along with non-linear and neural network models, as the best linear model (e.g., in task 1, SVC with mean accuracy/f1-score/ROC-AUC: 91.7%/0.819/0.926) may match or even outperform the performance of more complex models, especially if the task has a wide, rather than long data matrix, or if the classes are clearly separable.

Analyzing classifier performance purely using quantitative metrics provides only a narrow view, however. Our analysis reports additionally provide plots on class imbalance, input data distribution, and confusion matrices, all of which provide different insights into the experiment. Class representation in the dataset correlated with the sensitivity for each class in all three experiments, which the confusion matrices highlighted. The input data distribution additionally revealed that DizzyReg data in our study had a fundamental separation into two clusters (cf. UMAP embeddings in Figures 2–4). At least in task 1 this did not affect classification outcomes to match the clinical intuition, however, for future ML-based studies, this separation would need to be investigated further. Counteracting such a data separation, e.g., with input data transforms (54), or more advanced techniques like domain adaptation (55), could improve classification results further. As such, the results obtained through the base-ml tool provide not only information about which machine learning models to pursue further, but they also indicate starting points regarding the optimization of the input data with classical data science and statistical methods. For clinicians, an important part of the results are the most important features selected by the classifiers, which we present in an aggregated form using the proposed RAFI heuristic. These features will be discussed in more detail and put into a clinical context in section Clinical Implications.

The method presented in this work, and comprised in the base-ml tool have several noteworthy limitations. In general, base-ml is intended as a first screening tool for ML experiments, rather than as a complete ML solution that leads to a trained model for prospective studies and/or deployment. It has been shown previously that hyper-parameter optimization using nested cross-validation can lead to significant improvements of classification performance (6, 12, 13). In our study, while hyper-parameter tuning had no noticeable effects on Task 2, there were noticeable improvements in the average classification outcomes across all models in Tasks 1 and 3. Further, not only the models themselves have hyper-parameters, but every part of the ML pipeline in base-ml could be individually optimized further. This could include alternative input normalization strategies [e.g., power transforms (54, 56)] and imputation methods [e.g., kNN imputation or multiple imputation by chained equations, MICE (57, 58)] or the inclusion of feature selection methods (e.g., based on univariate hypothesis testing), all of which are important toward optimal classifier performance (9). A default treatment made in our experiments, for example, is to discard variables that were recorded for <50% of the population. In clinical practice, however, some variables may be missing because the according examinations or apparative tests were not ordered by the physician, maybe due to time, cost, lack of indication, or expected inefficacy toward diagnosis. In that case, individual rules for variable rejection, imputation and/or normalization may be necessary. For base-ml, we chose to avoid such in-depth treatment, in favor of an ease-of-use at the exploratory stage. However, base-ml is built on top of scikit-learn and already provides an interface to modern deep learning methods with skorch, and explainable AI solutions through Captum. This makes it easy to include many further methods for feature selection, imputation and normalization, as well as further classification explainable AI algorithms (32). However, at a certain level of complexity that aims at deployment rather than exploration, it is recommendable to consider more in-depth analyses and tool, ideally in close collaboration with data science and ML experts, and potentially starting off from insights obtained with base-ml. A particularly interesting avenue is the current research direction of Automated Machine Learning (AutoML), which aims at an optimization of the entire classification pipeline end-to-end (59). Importantly though, small to medium-size datasets might not provide enough data samples to train such complex pipelines. Until more cross-institutional vestibular registry datasets like DizzyReg come to existence, and with sufficient data to apply AutoML, the methods which we wrapped in base-ml and presented in this work still provide a solid starting point for ML-based analysis. As such, and for the time being, we believe these tools to be a valuable contribution for the vestibular research community.

### Clinical Implications

Clinical reasoning in the diagnostic differentiation of common vestibular disorders is based on a “mental aggregation” of information from patient characteristics (such as age and gender), symptom characteristics (namely quality, duration, triggers, accompanying symptoms), clinical examination (e.g., positioning maneuvers), and quantitative tests of vestibular function (such as vHIT, calorics) (16). It is an open and relevant question, whether ML-based methods are able to identify features from a multimodal vestibular patient registry, which resemble this clinical thinking and feature weighting. In the current study, we tested three clinical scenarios of different complexity on the DizzyReg database to further address this issue.

The first classification task represented two groups of patients suffering from chronic dizziness of almost diametrical etiology. In bilateral vestibular failure, imbalance can be directly assigned to an organic damage of vestibular afferents, which is accompanied by a low degree of balance-related anxiety (60, 61), while in functional dizziness the vestibular system is physiologically intact, but the subjective perception of balance is severely disturbed due to fearful introspection (62). It can be expected that ML-based algorithms will predominantly select features as most important for the segregation of both disorders, which represent either measurements of vestibular function or scales for anxiety and perceived disability. Indeed, the top 10 important features exactly meet this assumption with six of them reflecting low and high frequency function of the vestibular-ocular reflex (HIT left/right normal, vHIT gain left/right, bilateral vHIT normal, caloric response normal), and further three features healthy-related quality of life, depression and fear. Furthermore, age was an important differential feature, which is in good accordance to the fact that bilateral vestibular failure appears more frequently in older patients and functional dizziness in younger and mid-aged patients.

In the second classification task, two groups of patients with functional dizziness were compared, who were presumably very similar in their symptomatic presentation, but differed in the evolution of their symptoms: patients with primary functional dizziness, where chronic psychological stress or anxiety is the driving force, and patients with secondary functional dizziness, which develops after a preceding somatic vestibular disorders (e.g., BPPV) due to altered balance perception and strategies of postural control (8). Accordingly, top 10 features for classification included vestibular function tests (such as vHIT gain left/right and caloric response normal). The subtle differences between groups may speak for a partially recovered acute unilateral vestibulopathy or MD as some causes underlying secondary functional dizziness. Furthermore, symptom provocation by position changes in bed may point to BPPV as another vestibular disorder triggering secondary functional dizziness. This findings agree with previous literature (8). Interestingly, patients with primary functional dizziness had higher fear and depression scales, which may indicate a more intense psychological symptom burden. Indeed, previous studies have shown a psychiatric comorbidity in primary functional dizziness in 75 vs. 42% in secondary functional dizziness (63). The more frequent consumption of alcohol in primary functional dizziness may also show that those patients subjectively profit from its relaxing effects to a higher extent than patients with secondary functional dizziness, who have some degree of vestibular deficits, which may exacerbate on alcohol (e.g., partially compensated unilateral vestibulopathy or vestibular migraine).

The third classification task was designed to differentiate common episodic vestibular disorders like BPPV, MD, vestibular migraine and vestibular paroxysmia. Expectedly, a set of features was most indicative for BPPV, namely short attack duration and provocation by position changes. MD as compared to the other vestibular disorders was associated with the highest rate of pathological vestibular function tests (caloric test abnormal). It is well-known that long-standing MD can cause vestibular function deficits (64), while this is less frequent in vestibular migraine (65). The latter was associated with the highest frequency of headache and the youngest mean patient age, in accordance to literature (66). Vestibular paroxysmia was mostly defined by a short-symptom duration. The overall moderate accuracy for classification of the four episodic vestibular disorders can be explained by several factors: (i) one methodological explanation could be that this was a multi-class task, which is more challenging; (ii) despite the exhaustive history taking and examination details for patients recorded in DizzyReg, it is possible that not all relevant information is included. For example, systematic audiological test results are only available for patients with Menière's disease and vestibular migraine, but not for BPPV or vestibular paroxysmia. Therefore, audiological test results could not be generally included in the third classification task as a variable; (iii) there are potential overlaps of symptom characteristics and features. A prominent example is an overlap syndrome of MD and vestibular migraine, which could point toward a common pathophysiology (67); (iv) although the guidelines “International Classification of Vestibular Disorders (ICVD)” of the Barany Society give clear criteria for diagnosis mostly based on history taking, complex clinical constellations such as overlapping syndromes or atypical presentations appear regularly in the practice of a tertiary referral center, which may cause some difficulties in clear-cut classification. Limited classification accuracy may be partly explained by this selection bias, and further testing in primary care settings will be needed; (v) given the difficulty of task 3, the low ML classification performance is neither surprising nor a sign of a failure of ML classification approaches. Instead, our results suggest that ML algorithms, even given considerable data to learn from, may not automatically be able to solve difficult clinical tasks. The wide range of tuned ML algorithm performances presented by base-ml can reveal such difficulty better than a narrow selection of ML results without tuning; (vi) previous studies suggest that expert consensus may not always be unanimous, and may indicate the difficulty of patient diagnosis, despite clear guidelines and diagnostic criteria. For example, authors in (68) tried to validate diagnostic classifications through multi-rater agreement between several experienced otoneurological raters, and an acceptable consensus was achieved only in 62% of the patients. This study indicates that some diagnostic inaccuracy persists in the clinical setting, despite established international classification criteria. This could be taken as a further argument to augment clinical decision making by ML-based support systems.

## Conclusion

Analysis of large multimodal datasets by novel ML/MVA-methods may contribute to clinical decision making in neuro-otology. Important features for classification can be identified and aligned with expert experience and diagnostic guidelines. The optimal ML/MVA-method depends on the classification task and data structure. Base-ml provides an innovative open source toolbox to test different methods and clinical tasks in parallel. The multi-faceted presentation of results and explainable AI features, including an identification of clinically relevant features and their statistical analysis, enables clinicians to better understand ML/MVA outcomes, and identify avenues for further investigation. Future research needs to be extended to larger multicenter datasets and new data sources to improve the performance of automated diagnostic support tools.

## Data Availability Statement

The data analyzed in this study was obtained from the DSGZ DizzyReg, the following licenses/restrictions apply: The DSGZ provides application forms that must be completed before the data in the DizzyReg may be accessed. Please contact the DSGZ for more details on the application process. Requests to access these datasets should be directed to Ralf Strobl, ralf.strobl@med.uni-muenchen.de.

## Ethics Statement

The studies involving human participants were reviewed and approved by Institutional Review Board University Hospital Munich Ludwig Maximilian University Munich, Germany. The patients/participants provided their written informed consent to participate in this study.

## Author Contributions

GV, AZ, RS, and S-AA contributed to conception and design of the study and wrote the first draft of the manuscript. NN and EG contributed to study refinement. RS and GV organized the database. GV and S-AA developed base-ml and performed the data and statistical analyses. S-AA, AZ, NN, and EG provided funding. All authors contributed to the article and approved the submitted version.

## Conflict of Interest

The authors declare that the research was conducted in the absence of any commercial or financial relationships that could be construed as a potential conflict of interest.

## Publisher's Note

All claims expressed in this article are solely those of the authors and do not necessarily represent those of their affiliated organizations, or those of the publisher, the editors and the reviewers. Any product that may be evaluated in this article, or claim that may be made by its manufacturer, is not guaranteed or endorsed by the publisher.

## Figures and Tables

**Figure A1:**
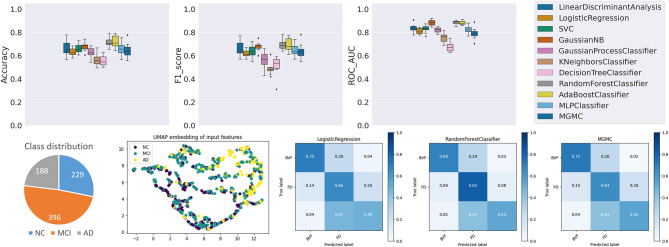
Results panel produced by base-ml for TADPOLE.

**Figure A2:**
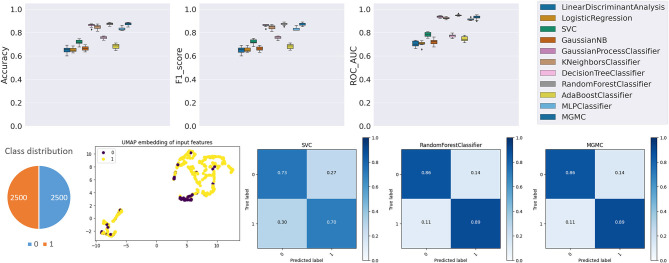
Results panel produced by base-ml for Synthetic data.

## Footnotes

^1^Base-ml source code and documentation: https://github.com/pydsgz/base-ml.

^2^Scorch source code and documentation: https://github.com/skorch-dev/skorch

^3^Captum source code and documentation: https://github.com/pytorch/captum

## Appendix

### Appendix A. Supplementary Experiments on TADPOLE Dataset

#### Data Description

TADOLE (69) is an ADNI-based dataset consisting of imaging-derived features and non-imaging features. The task is to classify whether observations at a baseline timepoint are from healthy normal controls (NC), patients with mild cognitive impairment (MCI), and Alzheimer's disease (AD). It consists of 813 instances (229 NC, 396 MCI, and 188 AD). Imaging features are computed using standard ADNI feature extraction pipelines.

#### Results and Discussion

We evaluated all models on this dataset as supplementary experiment to understand the strengths and limitations of our proposed model. For our purposes we only look at the F1-score, as this metric is more robust to class imbalance, which is present in TADPOLE. We observe that the best performing models are the hyper-parameter-optimized tree-based models such as Random Forest and AdaBoostClassifier. Furthermore, neural network based models such as MLPClassifier and MGMC yield comparable results but do not outperform other models. We also observe from the confusion matrices that the biggest source of error in most models is to distinguish patients with diagnosed AD from patients with MCI. Likewise, the confusion matrices reveal that models almost never mistake healthy controls with AD patients and vice-versa. Overall almost all models perform comparably, except notable mis-classification rates in KNeighborClassifier and GaussianProcessClassifier. Our obtained classification results of ~0.6–0.7 F1-score are in line with recent literature, e.g., our previous comparison of MGMC with regular machine learning classifiers [cf. results in (13), not yet computed with base-ml], or RF-based AD classification by Gray et al. (70).

### Appendix B. Supplementary Experiments on Generated Dataset

#### Data Description

To further illustrate the utility of base-ml, we created a synthetic dataset for a binary classification task. We generated 5,000 samples with 20 features of which 10 features are informative and the remaining 10 are uninformative using Scikit-learn (48) (using the built-in function < make_classification>). It is important to note that by design, this classification task has a non-linear separation boundary between the two classes and can therefore not be solved with high accuracy by linear classifier models.

#### Results and Discussion

As can be seen in Figure A2, most non-linear models based on neural networks and properly tuned tree-based models such as Random Forest could yield comparable performance. When looking at the classification accuracy of both MGMC and Random Forest, both perform nearly identically, and with the highest accuracies among all models. As expected, the linear models such as Logistic Regression and Linear Discriminant Analysis obtained the lowest classification accuracy. Overall, we observe that base-ml properly reflects the statistical properties and the difficulty of this artificial classification problem. The source data distributions are not simply separable by topology mapping (see UMAP embedding), and the separation is only resolvable by selected and properly tuned non-linear models – this characteristic would not have been detected by an analysis that was limited to linear models, or less suited non-linear models (e.g., for this dataset: Decision Tree Classifier or AdaBoost Classifier).

### Appendix C. Implementation Details: Hyperparameter Search Ranges

To have a more comparable analysis, we selected the best hyperparameters using the validation set, before reporting performance metrics on a with-held test-set (nested cross-validation). We do this by randomly searching the hyperparameter space for 100 iterations for every model and select the best hyperparameters which yields the best validation set classification performance.

For Logistic Regression we used the following hyperparameters (C: randint(1, 11); penalty: {“elasticnet”}, solver: {“saga”}, l1-ratio: uniform(0, 1));

Random Forest (max_depth: {3, None}; max_features: randint(1, 11); min_sample_split: randint(2, 11); bootstrap: {True, False}; criterion: {“gini”, “entropy”}, n_estimators: randint(5, 50));

K-Neighbors Classifier (n_neighbors: randint(3, 100); weights: {“uniform”, “distance”});

SVC (C: log_uniform(1e−6, 1e+6); gamma log_uniform(1e−6, 1e+6); degree: randint(1, 8), kernel: {“linear”, “poly”, “rbf”});

Decision trees (max_depth: {3, None}; max_features: randint(1, 11); min_samples_split: randin(2, 11); criterion: {“gini”, “entropy”};

Gaussian Process Classifier (kernel: {1*RBF(), 1*DotProduct(), 1*Matern(), 1*RationalQuadratic(), 1*WhiteKernel()};

AdaBoostClassifier: (n_estimators: {50, 60, 70, 80, 90, 100}, learning-rate: {0.5, 0.8, 1.0, 1.3});

GaussianNB (var_smoothing: logspace(0, 9, num=100));

Linear Discriminant Analysis (solver: {“svd”, “lsqr”, “eigen”}; shrinkage: numpy.arange(0, 1, 0.01));

MLP Classifier (learning-rate: {1e−1, 1e−2, 1e−3, 1e−4}; hidden-units: {32, 64, 128}, dropout probability: {0.0, 0.1, 0.2, 0.3, 0.4, 0.5});

MGMC ([cross-entropy, Frobenius-norm, Dirichlet-norm weighting]: uniform(0.001, 1000)).
